# Pilot Study on the Influence of Nutritional Counselling and Implant Therapy on the Nutritional Status in Dentally Compromised Patients

**DOI:** 10.1371/journal.pone.0147193

**Published:** 2016-01-28

**Authors:** Bernd Wöstmann, Teresa Simon, Monika Neuhäuser-Berthold, Peter Rehmann

**Affiliations:** 1 Department of Prosthodontics, Justus-Liebig-University, Giessen, Germany; 2 Department of Agricultural Sciences, Nutritional Sciences and Environmental Management, Institute of Nutritional Science, Justus-Liebig-University, Giessen, Germany; The Ohio State University, UNITED STATES

## Abstract

**Objectives:**

To investigate the impact of implant-prosthetic rehabilitation combined with nutritional counseling on the nutritional status of patients with severely reduced dentitions.

**Design:**

An explorative intervention study including an intra-individual comparison of 20 patients with severely reduced dentitions in terms of nutrition- and quality of life-related parameters recorded at baseline and at six and twelve months after implant-prosthetic rehabilitation.

**Participants:**

Twenty patients from the Department of Prosthetic Dentistry of Justus-Liebig University of Giessen, with an mean age of 63 years, who had fewer than ten pairs of antagonists.

**Measurements:**

The baseline data collection included dental status, a chewing ability test, laboratory parameters, anthropometric data (body mass index), energy supply, a 3-day dietary record, an analysis of the oral health-related quality of life (OHRQoL) with the OHIP-G14, the Mini-Mental Status (MMS) and Mini Nutritional Assessment (MNA). Six months after implantation and prosthetic rehabilitation, individual nutritional counseling was performed by a dietician. Data were again collected and analyzed. A final follow-up was conducted 12 months after prosthetic rehabilitation.

**Results:**

Despite the highly significant improvement in masticatory ability and OHRQoL after implant-prosthetic rehabilitation, no significant changes were observed regarding MNA, anthropometric data or energy supply. Except for cholinesterase (p = 0.012), ferritin (p = 0.003), folic acid (p = 0.019) and vitamin A (p = 0.004), no laboratory parameter changed significantly during the investigation period. In addition, no general significant differences were observed for nutrient intake or food choice.

**Conclusion:**

The present study does not confirm the assumption that the implant-prosthetic rehabilitation of patients with severely reduced residual dentitions with or without an individual nutritional counseling influences nutritional status.

## Introduction

Despite the extensive establishment of implant-supported prosthetic restoration, thus far, a comprehensive evaluation of its effectiveness, particularly with respect to the influence on the nutritional status and quality of life after implant-supported prosthetic treatment, has not been performed.

The correlation between current nutritional status and dental status has previously been discussed in several studies [1–8]. An impaired ability to chew has a negative effect on food selection and diet [1,2,6]. In addition, increasing tooth loss leads to a change in dietary composition [3,5,9]. In addition to gastrointestinal disorders, [10,11] an inadequate diet can result in malnutrition, with a prevalences of 0–10% for independent elderly individuals and 50% for geriatric acute or hospitalized patients [12–14].

The influence of an optimized dental status due to a positive dietary change depends on general health, socioeconomic status, individual dietary habits and condition of the masticatory system [15]. Food rejection is primarily due to masticatory disorders. Furthermore, reduced taste sensation or long-lasting adaption may require a rationalized nutrition plan [16]. Thus, improved nutritional behavior is not guaranteed after prosthetic and masticatory rehabilitation. An individually tailored nutrition intervention simplifies dietary changes for prosthetic rehabilitated elderly individuals [17].

The influence of prosthetic restorations on nutritional status has been previously discussed, particularly concerning complete and removable partial dental prostheses often also referred to as “removable partial denture” (RPD) [3,4,18]. In addition, various groups have investigated potential improvements in nutritional status and quality of life by both conventional and implant-supported dentistry [19–24]. Whether an implant-supported suprastructure supplying severely reduced dentition leads to an improved diet cannot currently be answered unequivocally in the literature. There is a lack of clinical studies containing before/after comparisons of implant-prosthetic treatments. Additionally, few studies have investigated blood parameters and nutrient intake over a period of several months [23,25,26].

This study investigated the impact of nutritional counseling on the nutritional status of patients with severely reduced dentitions after implant therapy in a pre-post design. The counseling aimed to help patients use their enhanced chewing efficiency to improve their personal diet. To the best of our knowledge, data on the effect of nutritional counseling on patient nutritional status after implant therapy are limited. This study was intended as a pilot study to identify possible marker variables.

The following null hypothesis was tested: nutritional counseling does not influence nutritional status, as assessed through nutritional blood-markers and body mass index (BMI) in patients with severely reduced dentitions after implant-prosthetic rehabilitation.

Furthermore, we analyzed a variety of typical blood and nutritional parameters to identify possible marker variables for a large-scale study.

## Methods

### Patients

**Fig 1:**
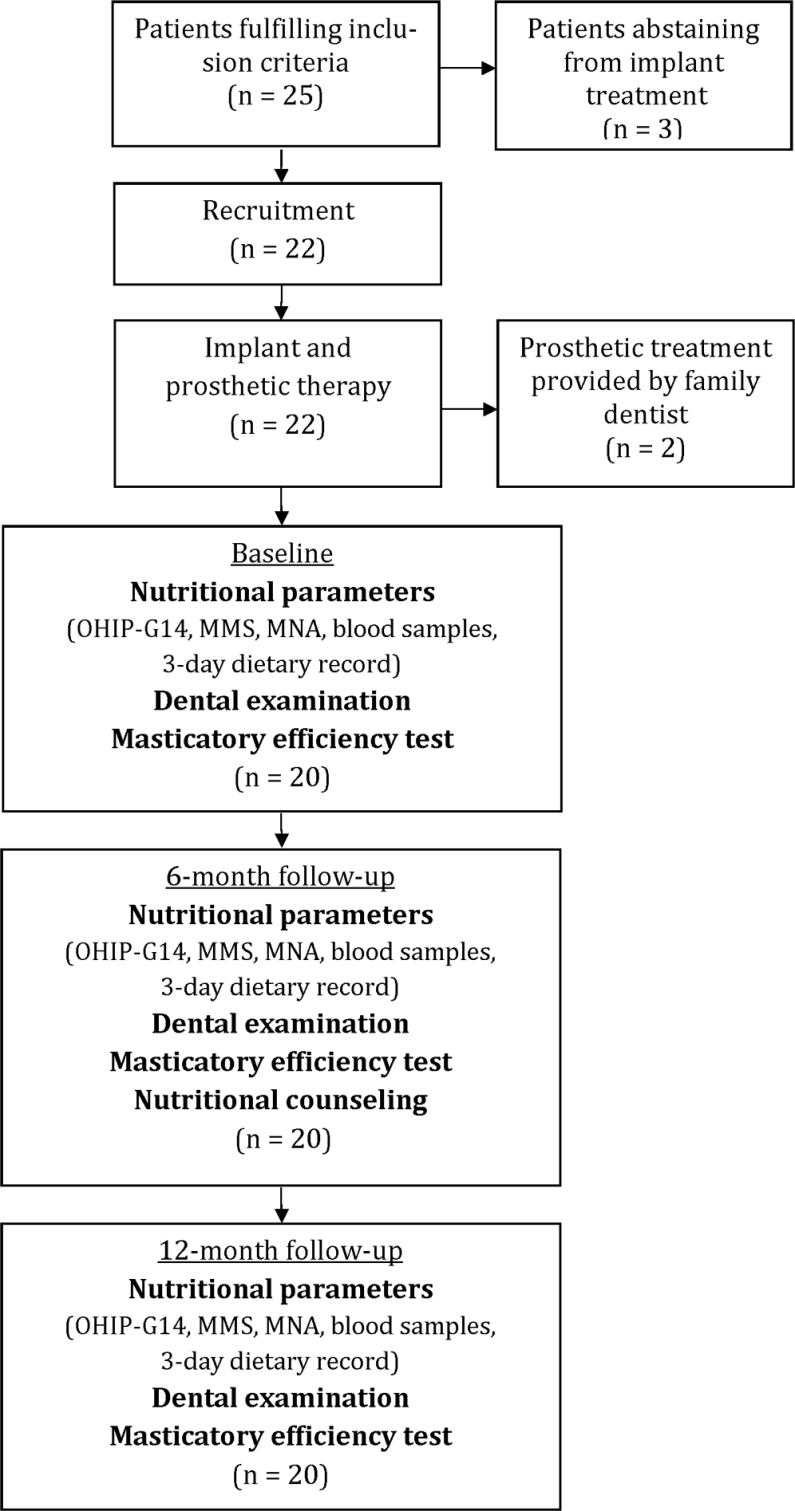
Flow of participants. Flow of participants through the intervention study and response rate of subjects.

Overall, 25 patients with fewer than ten pairs of opposing natural teeth (antagonists) and who were capable of feeding themselves were eligible and willing to participate. In all patients, a combined implant–prosthetic treatment with fixed or removable dental prostheses was planned. Patients addicted to medication, alcohol and/or drugs, suffering from malignant tumors or infectious diseases, undergoing radiation therapy, pregnant or breast-feeding or unwilling or incapable of consenting were excluded. Due to economic reasons, three patients completely abstained from the planned implant–treatment and could not be included. Thus, 22 subjects were recruited for the clinical trial (Fig 1). Informed consent was obtained from all participants in writing.

After the beginning of the study, two patients were additionally excluded because they opted for a prosthetic treatment by their family dentist.

The remaining 20 participants (Table 1) were treated at the Department of Prosthetic Dentistry of the Justus-Liebig University of Giessen between July 1^st^, 2009 and August 31^st^, 2012. There was no loss to follow-up at six and twelve months.

**Table 1:** Characteristics of the patients.

Gender	Age(Mean + SD Range)	Type of implant treatment	Number of occluding pairs before and after treatment (Mean + SD)	Number of implants (Mean + SD)
10 female	62.5 ± 7.79 years	12 RISDPs	7 ± 2 before implant treatment	7 ± 3 implants
10 male	50–76 year	8 FISDPs	12 ± 2 after implant treatment	

Characteristics of the patients included in the study (RISDP = Removable implant supported dental prosthese, FISDP = Fixed implant supported dental prostheses)

The study was approved by the Ethics Committee of the Justus-Liebig-University Giessen, Germany (Jan. 29^th^, 2008; Reg. No.: 181/07) and due to administrative delays registered in the German Clinical Trials Register on Dec 8^th^, 2009 (DRKS-ID: DRKS00000155). The authors confirm that all related trials for this intervention are registered.

### Methods

At the baseline, 6-month follow-up and 12-month follow-up examinations, patient dental status was assessed, and the following tests were performed:

- Mini Mental Status (MMS)[27] acc. to Folstein
- Mini Nutritional Assessment (MNA)[28]
- Oral Health Impact Profile (OHIP), which is the most frequently used assessment in dentistry to analyze Oral Health-related Quality of Life (OHRQoL)[29]. In the present study, the German version of the OHIP (OHIP G14) was used[30].
- Masticatory function test
  To evaluate masticatory efficiency, the method described by Wöstmann and Nguyen was employed. The patients were asked to chew a standardized cube of carrot (2 cm x 2 cm x 1 cm) within 45 seconds into pieces as small as possible without swallowing any part of the carrot. The carrot pieces were collected in a Petri dish. Then, the degree of the comminution was evaluated visually by comparison with a reference scale (level 1 = fine; level 6 = impossible)[31].

Additionally, 17.7 ml of blood was taken from each patient to determine the serum values of hemoglobin, iron, total protein, albumin, pre-albumin, cholinesterase, HDL/LDL, triglycerides, cholesterol, ferritin, zinc, beta carotene, vitamins A, B12, C, and E and folic acid. All blood samples were taken between 8 am and 9 am. For sample collection, all patients fasted for at least 12 hours.

Anthropometric data (body mass index), energy supply, a 3-day dietary record, and an additional questionnaire determining dietary behavior were obtained. Both questionnaires were also used in the long-term GISELA study[32] and have been previously validated.

Six months after implant therapy and prosthetic rehabilitation with a minimum of 10 occluding pairs, individually tailored nutritional counseling was performed by a dietician at the Department of Agricultural Sciences, Nutritional Sciences and Environmental Management, Institute of Nutritional Science, Justus-Liebig University, Giessen, Germany. Individual counseling was based on the 3-day dietary record and dietary behavior questionaire,[32] which were completed by the patients in advance.

### Statistical analysis

All blood parameters and ordinal data (MNA, masticatory efficiency test, and OHIP-G14) were subjected to a Wilcoxon matched-pairs test (p = 0.05). To identify significant group differences, the Mann-Whitney test was used (p = 0.05). As the study was intended as a pilot it was decided to assume a change in weight of about 1kg in an average subject (1.75 m / 70 kg) as a basis for power calculation. Under this assumption a sample size of 18 (change in BMI: 0.35, std 0.5) was calculated for a desired power of 0.08 and a significance level of 0.05.

All data analyses were performed with the software package IBM SPSS Statistics 20 SPSS (IBM, Armonk, NY, USA).

## Results

**Fig 2:**
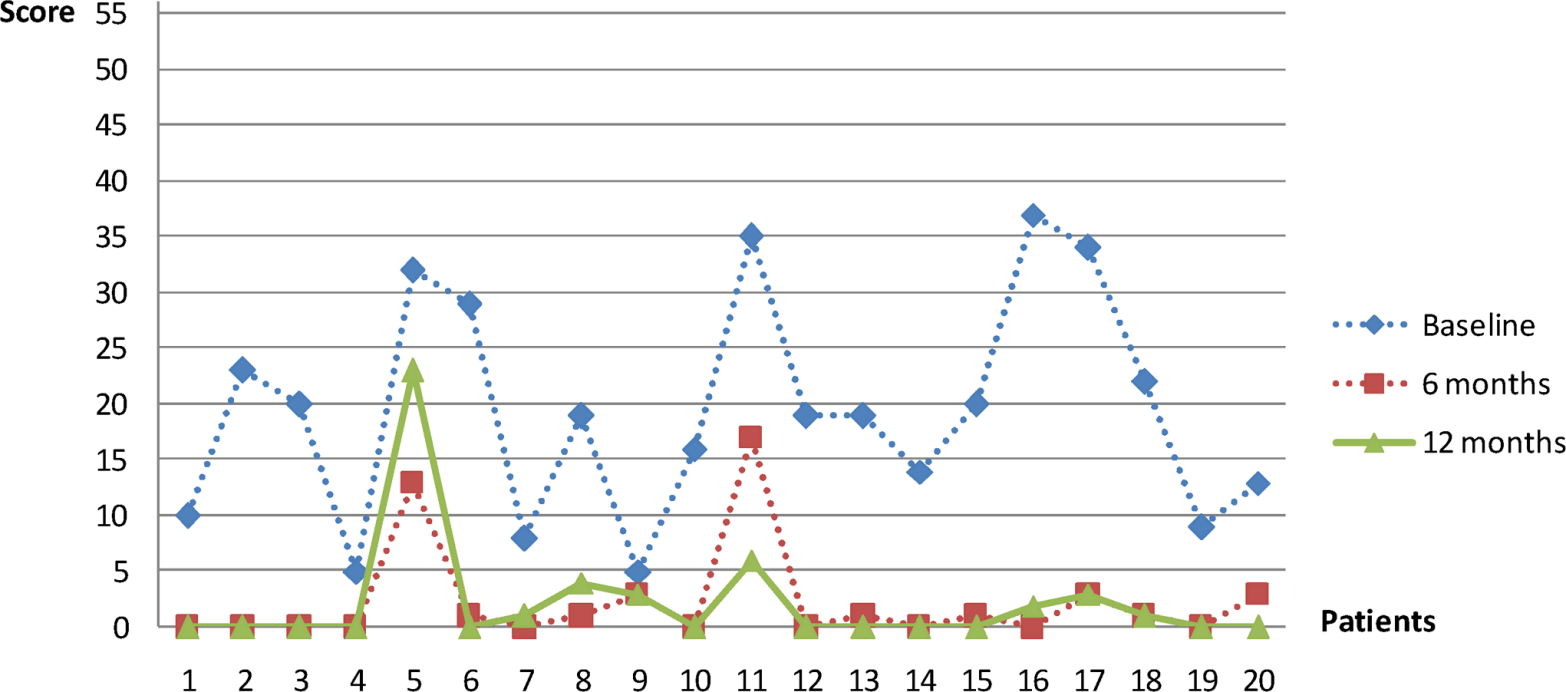
OHIP-G14 score. OHIP-G14 score at baseline and at 6 and 12 months following implant-prosthetic rehabilitation.

A significant improvement in OHRQoL after implant-prosthetic rehabilitation was observed (p<0.001) (Table 2). All 20 participants had a lower total OHIP-G14 score at the 6-month follow-up compared with baseline (Fig 2).

**Table 2:** Changes in OHIP-G14, masticatory efficiency, MNA, BMI and energy supply.

	Time point	Mean + SD	Difference	P value
**OHIP- G14**	Baseline	19.45 ± 9.90	-	-
	6 months	2.20 ± 4.55	-17.25 ± 8.63	**< 0.001**
	12 months	2.15 ± 5.19	-0.05 ± 3.61	0.471
**Masticatory**	Baseline	3.80 ± 1.99	-	-
**efficiency**	6 months	1.95 ± 1.36	-1.85 ± 1.90	**< 0.001**
**Score**^1^	12 months	1.95 ± 1.36	0.00 ± 0.79	1.000
**MNA**	Baseline	14.6 ± 3.0	-	-
	6 months	15.1 ± 3.6	0.5 ± 4.1	0.362
	12 months	14.7 ± 3.2	-0.4 ± 4.1	0.893
**BMI**	Baseline	26.8 ± 4.9	-	-
	6 months	27.1 ± 4.8	0.3 ± 1.0	0.148
	12 months	27.2 ± 5.0	0.1 ± 0.6	0.559
**Energy (kcal)**	Baseline	2137.53 ± 802.88	-	-
	6 months	2192.46 ± 631.53	54.93 ± 823.08	0.769
	12 months	2059.23 ± 873.09	-133.24 ± 810.20	0.471

Changes in OHIP-G14, masticatory efficiency, MNA, BMI and energy supply at baseline and at 6 and 12 months following implant-prosthetic rehabilitation.

^1^ 1: very high 2: high, 3: average, 4: reduced, 5: low, 6: poor masticatory efficiency

A significant improvement in chewing efficiency was observed six months after implant-prosthetic therapy (p<0.001). No subject exhibited decreased chewing efficiency. No further improvements from the 6- and 12-month follow-up were observed (p>0.05) (Table 2).

All patients had MMS scores above 27. No changes were observed during the observation period. The mean MNA increased only slightly from 14.6 ± 3.0 (baseline) to 15.1 ± 3.6 at the 6-month follow-up and 14.7 ± 3.2 at the 12-month follow-up (p>0.05) (Table 2). In addition, no significant changes were observed in terms of anthropometric data (BMI) or energy supply (p>0.05).

With the exceptions of cholinesterase (p = 0.012), ferritin (p = 0.003), folic acid (p = 0.019) and vitamin A (p = 0.004), no laboratory parameter changed significantly during the investigation period (Table 3). No change in nutrient intake or food choice (p>0.05) was observed (Tables 4 and 5). Beta-carotene, iron, zinc and carbohydrates decreased, whereas cholesterol and retinol intake increased.

**Table 3:** Plasma biomarkers.

Plasmabiomarkers		Time point		Difference 0–6 m p value	Difference 6–12 m p value
	Baseline	6 months	12 months		
**Hemoglobin [g/l]**	144.30 ±	147.85 ±	147.65 ±	3.55 ± 14.33	-0.20 ± 8.17
	20.44	14.36	10.87	0.527	0.837
**Iron [μg/dl]**	86.10 ±	90.95 ±	97.60 ±	4.85 ± 31.57	6.65 ± 43.71
	33.91	31.55	32.29	0.338	0.852
**Zinc [μg/dl]**	90.80 ±	83.50 ±	85.52 ±	-7.31 ± 23.53	2.63 ± 17.05
	16.30	16.15	11.49	0.322	0.722
**Total protein [g/l]**	72.30 ±	72.45 ±	72.10 ±	0.15 ± 3.57	-0.35 ± 2.23
	3.01	4.43	3.01	0.757	0.542
**Cholinesterase [U/l]**	9396.00 ±	9875.95 ±	9920.40 ±	480.00 ± 919.20	44.45 ± 902.58
	2082.48	2153.29	2057.51	**0.012**	0.794
**Cholesterol [mg/dl]**	216.85 ±	212.40 ±	210.45 ±	-4.45 ± 21.47	-1.95 ± 27.93
	37.51	40.48	41.87	0.350	0.455
**Triglycerides [mg/dl]**	114.45 ±	116.65 ±	109.00 ±	2.20 ± 41.11	-7.65 ± 35.03
	46.70	69.68	55.56	0.794	0.305
**HDL [mg/dl]**	59.85 ±	57.45 ±	59.35 ±	-2.40 ± 7.94	1.90 ± 6.80
	15.63	15.31	16.63	0.129	0.230
**LDL [mg/dl]**	141.70 ±	140.50 ±	140.55 ±	-1.20 ± 17.77	0.05 ± 29.28
	36.49	40.17	44.06	0.926	0.668
**Albumin [g/l]**	44.2 ±	44.5 ±	44.4 ±	0.3 ± 2.1/	-0.1 ± 1.6
	2.2	2.7	1.8	0.457	0.904
**Prealbumin [g/l]**	0.27 ±	0.28 ±	0.28 ±	0.01 ± 0.03	-0.02 ± 0.04
	0.05	0.06	0.05	0.186	0.972
**Ferritin [ng/ml]**	103.95 ±	129.80 ±	131.85 ±	25.85 ± 47.71	2.05 ± 38.62
	96.37	133.94	124.89	**0.003**	0.334
**Folic acid [ng/ml]**	12.37 ±	12.95 ±	10.66 ±	0.57 ± 4.64	-2.47 ±4.60
	6.80	6.90	5.97	0.670	**0.019**
**Vit. B12 [pg/ml]**	353.00 ±	351.05 ±	362.75 ±	-1.95 ± 78.63 /	11.70 ± 78.07 /
	112.73	143.12	124.74	0.588	0.341
**Vit. A [μg/dl]**	69.66 ±	60.73 ±	62.51 ±	-9.75 ± 11.59	1.23 ± 11.34
	10.38	12.48	8.10	**0.004**	0.541
**Vit. E [μg/dl]**	1586.60 ±	1456.00 ±	1532.30 ±	-53.82 ± 279.60	104.20 ± 210.10 /
	355.75	233.09	204.93	0.619	0.169
**Beta-carotene [μg/dl]**	39.68 ±	43.50 ±	183.37 ±	6.65 ± 25.67	118.06 ± 281.35
	23.23	32.38	307.18	0.277	0.129

Plasma biomarkers at baseline and at 6 and 12 months following implant-prosthetic rehabilitation

**Table 4:** Nutrient intake.

Nutrients		Time point		Diff. 0–6 m	Diff. 6–12 m
	Baseline	6 months	12 months	p value	p value
**Cholesterol [mg/d]**	285.03 ±	293.59 ±	316.11 ±	8.56 ± 151.75	22.52 ± 152.43
	138.97	104.07	153.80	0.601	0.455
**beta-Carotene [μg/d]**	3995.20 ±	3902.54 ±	3724.00 ±	-92.66 ± 3519.71	-178.54 ± 1567.24
	3160.78	4460.34	4587.97	0.526	0.478
**Iron [μg/d]**	13326.56 ±	13366.81 ±	12611.87 ±	40.26 ± 5892.09	-754.95 ± 4572.52
	5032.13	5325.17	4874.24	0.970	0.502
**Carbohydrate [mg/d]**	254179.85 ±	259521.33 ±	234241.87 ±	55.93 ± 823.08	-133.24 ± 810.20
	105835.64	76565.05	92310.33	0.601	0.179
**Retinol [μg/d]**	434.06 ±	491.54 ±	764.26 ±	57.47 ± 303.95	272.72 ± 1577.45
	196.38	272.03	1534.43	0.765	0.279
**Vit. B12 [μg/d]**	6.38 ±	5.73 ±	6.01 ±	-0.65 ± 4.49	0.28 ± 5.06
	3.98	2.52	4.69	0.737	0.627
**Vit. C [μg/d]**	117556.69 ±	130136.26 ±	111710.84 ±	12579.60 ± 58401.82	-18425.42 ± 69328.63
	81350.72	121129.34	94145.89	0.765	0.263
**Vit. E [μg/d]**	13014.17 ±	13605.18 ±	12305.49 ±	591.01 ± 7596.71	-1299.69 ± 6681.07
	9460.67	8328.52	7955.50	0.575	0.601
**Zinc [μg/d]**	12505.62 ±	12123.50 ±	11325.80 ±	-382.12 ± 6279.81	-797.71 ± 4790.12
	5590.94	4125.52	4015.70	0.881	0.575

Nutrient intake at baseline and at 6 and 12 months following implant-prosthetic rehabilitation

**Table 5:** Food selection.

Food		Time point		Diff. 0–6 m / p value	Diff. 6–12 m /p value
	Baseline	6 months	12 months		
**Bread and bakery products**	157.92	181.57	199.5	23.64 ± 126.40 / 0.360	17.93 ± 151.51 / 0.550
**Fish**	18.49	18.09	18.18	0.40 ± 48.35 / 0.779	-0.08 ± 37.44 / 0.806
**Meat**	106.54	99.23	80.5	7.31 ± 95.13 / 0.845	18.73 ± 77.86 / 0.223
**Meat products (e.g., sausages)**	35	26.67	24	8.33 ± 54.28 / 0.842	2.67 ± 35.18 / 0.348
**Vegetable**	130.67	160.93	147.95	-30.26 ± 155.81 / 0.867	12.98 ± 85.41 / 0.455
**Potatoes and potato products**	91.51	74.09	85.67	17.42 ± 106.03 / 0.112	-11.58 ± 96.59 / 0.409
**Cheese, quark**	46.21	44.25	43.09	1.96 ± 38.53 / 0.938	1.17 ± 38.93 / 0.875
**Milk**	178.79	178.73	179.74	0.06 ± 108.45 / 0.856	-1.01 ± 162.36 / 0.808
**Noodles, rice, etc.**	71.07	67.54	69.36	3.54 ± 62.51 / 0.466	-1.82 ± 89.65 / 0.913
**Fruit**	186.65	237.16	192.63	-50.51 ± 136.69 / 0.157	74.53 ± 188.31 / 0.127
**Salads**	86.33	92.42	111.98	-6.08 ± 205.72 / 0.875	19.57 ± 280.21 / 0.906
**Confectionery**	87.12	72.73	30.06	-14.38 ± 68.66 / 0.387	-42.67 ± 103.88 / **0.011**

Food selection at baseline and at 6 and 12 months following implant-prosthetic rehabilitation

## Discussion

This study was intended as a pilot study to identify possible blood and nutritional parameters suitable for a larger scale study on the effect of implant treatment on patient nutritional status. Thus, many parameters typically considered in a nutritional context were investigated. However, none of the 26 blood parameters exhibited potential for use as a marker variable. Due to the enormous costs of laboratory analysis of blood samples, only a limited number of patients were included, which is a shortcoming of this study. However, after baseline, no patients were lost to follow-up. The overall high cost of the implant treatment must be regarded as a potential source of bias as primarily patients with higher socio-economic status opt for implant treatment and thus where eligible for this study. However, these patients tend to be better nourished[15].

The most significant differences with regard to OHIP-G14 were observed between baseline and the second examination (p<0.001), indicating improvement in OHRQoL immediately following implant-prosthetic rehabilitation and an increase in antagonistic pairs. This improvement was observed in every patient. The significantly increased chewing efficiency was correlated with these parameters. Our results support those of Inukai et al. [33] and Brennan et al. [1].

A range of publications have addressed whether implant-prosthetic rehabilitation influences OHRQoL [21,22,34–37]. Compared with conventional dental prostheses, OHRQoL increased significantly after the implant-supported prosthetic restoration. Group differences were strongly observed for comfort and stability [22,37].

In concordance with the previous literature, these results demonstrate that implant-prosthetic rehabilitation commonly leads to enhanced masticatory efficiency [23,24,26,38–40]. In addition to a masticatory efficiency test, other technical measures can assess chewing efficiency. Awad et al. [26] used questionnaires to determine chewing efficiency among middle-aged edentulous patients. Compared with visually evaluated techniques (e.g., masticatory efficiency tests with carrots or artificial test food), questionnaires and food records do present a disadvantage regarding objectivity.

MNA is a popular instrument for determining potential malnutrition in the current literature [39,41–44]. Concerning the current results, no changes were observed throughout the study period. This result can primarily be explained by the fact that most participants did not exhibit indications of malnutrition or were classified as being at risk of malnutrition. Thus, no substantive improvements could have been expected.

In the present study, no significant changes were observed regarding anthropometric data (BMI) or energy supply (p>0.05). A trend towards reduced mean calorie intake was observed. Muller et al. [16] and Morais et al. [23] demonstrated a missing correlation between the insertion of dental implants and increasing anthropometric data (BMI) compared with conventional dental prosthesis patients.

During the investigation period, no laboratory parameter significantly changed, except for cholinesterase, ferritin, folic acid and vitamin A. Aside from these parameters, albumin and prealbumin are the most-studied blood parameters for assessing dietary status [23,26]. The reason for these constant values relates to a constant good nutritional status among the clientele and the long half-life of albumin, which is approximately 20 days [45]. One advantage of the present study design is the repeated analysis of all blood parameters throughout the observation period.

Considering the changes for nutrient intake and food choice, it should be noted that food selection is heavily impacted by socio-economic status and individual habits and tastes [46,47]. A non-significant decrease in fiber consumption was observed, which corresponds to the findings of Moynihan et al. [25]. However, fruit and vegetable intake increased slightly compared with the baseline level. These dietary habit changes are due to masticatory improvements (cf. Morais et al. [23]). Occasionally, a few signs of improved diet could be identified after dietary intervention. In general, food awareness increased in the clientele after the tailored diet plan; unfortunately, these changes were not significant.

## Conclusions

Collectively, the present study does not confirm the assumption that implant-prosthetic rehabilitation of patients with severely reduced residual dentitions with or without an individual nutritional counseling influences nutritional status. Of the blood parameters investigated, no parameter had potential as a specific marker. However, our results provide strong indications of a direct impact on OHRQoL and chewing ability among implant-rehabilitated patients, which confirms the functional advantages of implant prosthodontics.

## Supporting Information

S1 File Study protocol. (PDF)

S2 File Trend Statement. (PDF)

S3 File SPSS-Files containing the underlying data. (RAR)
