# Supplementary material for: Pilot Study on the Influence of Nutritional Counselling and Implant Therapy on the Nutritional Status in Dentally Compromised Patients
Source: PLoS One. 2016 Jan 28;11(1):e0147193. doi: 10.1371/journal.pone.0147193 (PMC4731208; doi:10.1371/journal.pone.0147193)
Supplement: S1 File — (PDF) [file pone.0147193.s001.pdf]

D. Genaues klinisches Studienprotokoll mit Biometrie:

D. Accurate clinical trial protocol with biometrics :

D.1 STUDIENVERLAUF

STUDY COURSE

Die Patienten werden im Rahmen der interdisziplinären Implantatsprechstunden der Abteilungen Zahnärztliche Prothetik und Mund-, Kiefer- und Gesichtschirurgie rekrutiert und die Indikation zur kaufunktionellen Rehabilitation mit implantatgetragenem Zahnersatz gestellt.
Für alle Patienten werden Anamnesebögen nach MNA und OHIP (Oral Health Impact Profile) angelegt und ausgewertet. Zusätzlich wird ein Kaufunktionstest (Zerkleinerung von Karottenstücken, visuelle Auswertung) vorgenommen und ein Ernährungsassessment durchgeführt. Letzteres erfolgt mit einem geschlossenen 3-Tage-Schätzprotokoll zur Erfassung der Lebensmittel- und Nährstoffaufnahme sowie mit einem zusätzlichen Fragebogen zur Ermittlung des Ernährungsverhaltens. Die allgemeinmedizinische und zahnärztliche Anamnese so wie die Beurteilung der dentalen/ parodontalen Situation und des vorhandenen Zahnersatzes wird durchgeführt. Die Entnahme der Blutproben für die laborchemischen Untersuchungen erfolgt durch die Prüfärzte oder die jeweiligen behandelnden Ärzte. Die Blutprobenentnahme erfolgt nüchtern. Diese Zielparameter werden vor Beginn der Behandlung, ½ und ein Jahr nach Eingliederung des implantatgestützten Zahnersatzes erhoben (Abb. 1). Nach 6 Monaten erfolgt eine Ernährungsberatung.

Patients will be recruited from the patients of the departments of Prosthodontics and Oral and Maxillofacial Surgery.
For all patients medical history sheets are recorded and an MNA and OHIP assessment carried out. In addition, a masticatory function test (shredding carrots pieces, visual evaluation) is performed and a nutritional assessment conducted. The latter is carried out with a closed 3-day estimation protocol to capture the food and nutrient intake, and with an additional questionnaire to determine the nutritional behavior. The general medical and dental history as well as the assessment of dental / periodontal situation and the existing dentures is performed. The collection of blood samples for laboratory chemical tests carried out by the investigators or the respective doctors. The blood sampling is done sober. These target parameters before starting treatment, ½ and one year after inclusion of implant dentistry raised (Fig. 1). After 6 months there is a nutritional advice.

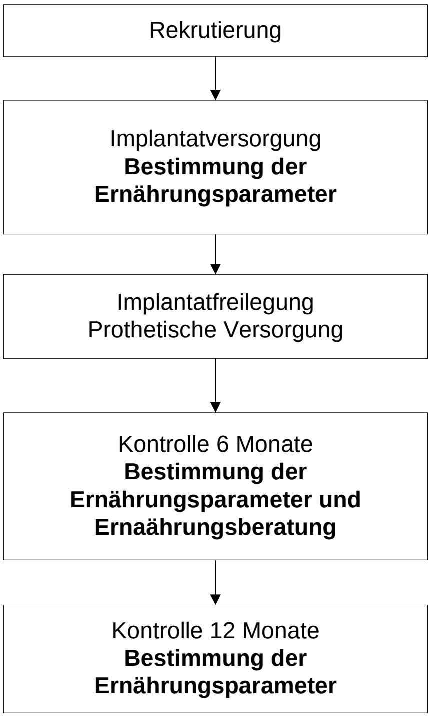

English translation see manuscript

Abb. 1: Übersicht über den geplanten Studienablauf
Overview of the planned study

D.2 HÄUFIGKEIT DER STUDIENBEDINGTEN ARZTBESUCHE
FREQUENCY OF STUDY RELATED VISITS

There are no additional visits for the patient. The study-related examinations are performed as part of routine visits and necessary follow-up examinations of the implants.

D.3 ABBRUCHKRITERIEN
Criteria for premature termination of the study

- Für den einzelnen Patienten / for a single patient
  - Verlust aller gesetzten Implantate / loss of all implants
  - Nachträgliches Bekanntwerden eines Ausschlusskriteriums
  - Subsequent appearance of an exclusion criterion

- Für die gesamte Studie / for the entire study

  - keine; da ein Auftreten von schweren unerwünschten Ereignissen im Sinne der ISO/EN 14155 Teil 1 und 2 für klinische Studien nicht zu erwarten ist, da es sich bei

der Implantattherapie um ein seit Jahren routinemäßig angewandtes Verfahren handelt.

- none ; since an occurrence of serious adverse events in accordance with the ISO / EN 14155 Parts 1 and 2 is not expected for this trial because it is a for years routinely used method for implant therapy.

D.4 ZUSÄTZLICHE BEHANDLUNGEN
ADDITIONAL TREATMENT

Im Rahmen der Einheilung von enossalen Implantaten kann es zu einem Implantatverlust und Misserfolg der Behandlung kommen. Aktuelle Verlustraten liegen in der Literatur zwischen 1- 5%. Ist bei einem Studienpatient zu einem Implantatverlust gekommen, ist wie bei jedem anderen Patienten im Einzelfall eine erneute Implantation oder bei Patienten mit mehreren Implantaten eine prothetische Versorgung auf weniger Pfeilern zu erwägen. Der Implantatverlust und die Folgen für die prothetische Suprakonstruktion werden im Studienprotokoll vermerkt.

During the healing phase implants may be lost resulting in a failure of the treatment. Current loss rates are reported in the literature between 1- 5 %. In case of implant loss either the placement of a new implant or – in patients with multiple implants - a prosthetic restoration on the remaining implants is considered. The implant failure and the consequences for the prosthetic superstructure are noted in the study protocol.

D.5 INTERVENTIOSSCHEMA
INTERVENTION PROTOCOL

Nach Planung der Implantation mit Hilfe von Röntgendiagnostik und Modellanalyse können in einem operativen Eingriff die enossalen Implantate inseriert werden. Die Markierung zur Implantatlage werden mit einer nach prothetischem Set-up hergestellten Bohrschablone mit dem Rosenbohrer und Pilotbohrern festgelegt. Nach standardisiertem Verfahren werden nun die enossalen Implantate eingebracht. Die Einheilzeit ohne Belastung der Implantate beträgt 4-6 Monate, danach werden sie in einem zweiten Eingriff freigelegt und können prothetisch versorgt werden.
Nach einer Tragedauer des neuen Zahnersatzes von 6 Monaten erhalten die Patienten im Anschluss an die erste Kontrolluntersuchung eine Ernährungsberatung, die den Zweck verfolgt, den Patienten in die Lage zu versetzen, die gestiegenen Möglichkeiten der Nahrungszerkleinerung ernährungsphysiologisch sinnvoll zu nutzen.
After treatment planning by means of X-ray diagnostics and model analysis, the intra-osseous implants are inserted. Implant positions are determined using a template prepared by diagnostic set-up. Implants will be inserted using the related protocol. The healing period without loading of the implants is 4-6 months, afterwards implants are revisited in a second intervention as prescribed in the respective implant protocol. Afterwards the planned prosthesis can be fitted
After a six 6-month period patients were seen for a check-up of implants and prostheses and received a nutrition counseling with the aim to enable the patients to take advantage of their increased chewing capabilities for the comminution of food.

D.6 EINSCHLUSSKRITERIEN / see M&M section

Es sollen teilbezahnte Patienten in die Studie eingeschlossen werden, die

- über weniger als 10 natürliche Antagonistenpaare verfügen,
- mit enossalen Implantaten zum Ersatz der fehlenden Zähne versorgt werden sollen,

- selbst zur Nahrungsaufnahme in der Lage sind.

D.7 AUSSCHLUSSKRITERIEN / see M&M section

Ausgeschlossen von der Studie sind solche Patienten, die

- medikamenten- alkohol- und / oder drogenabhängig sind,
- an malignen Tumoren leiden,
- an Infektionskrankheiten (HIV, Hep. A,B,C) leiden,
- parenteral ernährt werden oder zur Nahrungsaufnahme auf Hilfe angewiesen sind (füttern),
- an sich einer Strahlentherapie unterziehen- oder unterzogen haben,
- schwanger sind oder stillen,
- nicht bereit oder unfähig sind, ihre Einwilligung zur Teilnahme an der Studie zu geben.

D.8 ZIELVARIABLEN

TARGET VARIABLES

Wesentliche Parameter: / primary parameters

- Mini Nutritional Assessment (beinhaltet Body Mass Index)
- Albumin
- Vitamin C

Außerdem sollen folgende Parameter ermittelt werden: / secondary parameters

- Prä-Albumin, Mikronährstoffe: Vitamin A, B12, E, Folsäure (in Erythrozyten), beta-Carotin, Zink (Vergleichbarkeit mit Sahyoun [NHANES III Daten]), Hämoglobin, Eisen (Ferritin), Triglyceride, Gesamt-, HDL- und LDL-Cholesterin (British Diet and Nutrition Survey) [34],
- pre - albumin , micronutrients : vitamins A , B12 , E , folic acid (in red blood cells) , beta - carotene , zinc (comparability with Sahyoun [NHANES III data]) , hemoglobin , iron (ferritin) , triglycerides , total , HDL and LDL cholesterol (British Diet and Nutrition Survey)

- Tägliche Lebensmittel- und Nährstoffzufuhr
- Daily food and nutrient intake

- Ernährungsverhalten
- Nutritional behavior

- Mundgesundheitsbezogene Lebensqualität: OHIP G 14
- oral health -related quality of life :

- Kaufähigkeit: Ergebnis Kaufunktionstest (Wickop, Ha-Thi, Ngyuen, Kolb, Wöstmann)
- Masticatory function test: results

Deskriptiv zur Beschreibung des Patientenkollektivs: / description of the patients

- Zahnärztliche Parameter: Zahnstatus, Zustand der prothetischen Versorgung, Parodontaler Screening Index (PSI)
- • Dental parameters : dental status , condition of the prosthetic restoration , Periodontal Screening Index (PSI)

D.9 METHODEN GEGEN BIAS / methods against Bias

Da die Patienten aus zwei Zentren (Abteilung Prothetik und Mund-, Kiefer– und Gesichtschirurgie) rektrutiert werden, ist ein breites Spektrum von Patienten mit reduziertem Zahnstatus gegeben. Es werden alle Patienten, die sich in den beiden Implantatsprechstunden vorstellen und den Einschlusskriterien entsprechen in die Untersuchung mit einbezogen. Eine Stratifizierung erfolgt bezüglich des Alters.
Since the patients from two centers (Department of Prosthodontics and oral and maxillofacial surgery) are recruited, a wide range of patients is to be expected. All will be included in the study; stratification is done in terms of age.

Die chirurgische und prothetische Versorgung der Patienten erfolgt nach einem klinisch erprobten standardisierten Verfahren. Die zu ermittelnden Parameter werden standardisiert in nur einem Labor bestimmt.
The surgical and prosthetic procedures are based on a clinically proven standardized procedure. Blood parameters will be only analyzed in a single laboratory.

D.10 FALLZAHL- UND POWERBERECHNUNG Powercalculation

Mit Hilfe der wenigen in der Literatur zur Verfügung stehenden Informationen wurde mittels Power/Fallzahlberechnungen für die wesentlichen Parameter MNA, Albumin und Vitamin C ein Stichprobenumfang von N = 85 Probanden ermittelt, damit klinisch relevante Effekte aufgezeigt werden können.
With the help of the few available in the literature information sample size of N = 85 subjects was determined by power / sample size calculations for the most important parameters MNA , albumin and vitamin C , to identify clinical relevant effects.

Die in dieser explorativen Vorabstudie zu ermittelnden Daten sollten es erlauben, eine anschließende randomisiert kontrollierte Studie zu planen.
In this pilot study data to be determined are intended to be used for planning a subsequent randomized controlled trial .

Compliance - Loss to follow up

Erfahrungsgemäß ist die Compliance bei implantologisch versorgten Patienten sehr hoch, da diese regelhaft ein sehr hohes Interesse daran haben, den langfristigen Erfolg der primär aufwendigen Behandlung sicherzustellen. Aus diesem Grund wird ein Loss to follow up von ca. 5 % angenommen. Dieser Wert entspricht auch den Beobachtungen im Rahmen der DFG- geförderten Studie zur verkürzten Zahnreihe.
Experience has shown that compliance is very high in patients treated with implants, as they usually have a very high interest in ensuring the long-term success of the treatment. For this reason, a Loss to follow-up of about 5% is assumed. This value corresponds to the observations in the context of the DFG -funded study on the shortened dental arch.

D.11 DURCHFÜHRBARKEIT DER REKRUTIERUNG
FEASIBILITY OF RECRUITMENT

Die Gesamtzahl aller Implantatpatienten liegt bei ca. 70 Patienten mit 330 inserierten Implantaten pro Jahr. Im Rahmen der Implantatsprechstunde können aus diesem Patientengut ca. 60 Patienten pro Jahr rekrutiert werden, die den Einschlusskriterien entsprechen. Zusätzlich können in den ersten beiden Studienquartalen Studienteilnehmer aus der Gruppe derjenigen Patienten rekrutiert werden, bei denen bereits Implantate gesetzt wurden, die aber noch nicht freigelegt sind (ca. 25 Patienten). Damit ist die oben genannte Fallzahl realistisch rekrutierbar.

The total number of implant patients in our Hospital is about 70 patients with 330 implants inserted each year. Out these patients approximately 60 patients per year meet the inclusion criteria. In addition, study participants can be recruited from the group of those patients in the first two quarters of study, where the implants have already been inserted, but are still in the healing period. Thus, the above-mentioned number of cases seems to be realistic.

Die biometrische Betreuung bezüglich Methodik, Studiendesing, Randomisierung und Auswertung erfolgt durch AG Medizinische Statistik des Instituts für Medizinische Informatik der Universitätsklinik Gießen (Leiter: Dr. R.-H. Bödeker).

Die Hauptfragestellung bezieht sich auf die Gesamtbehandlung (von Behandlungsbeginn t_0 bis zum letzen Recall t_2 nach 12 Monaten): Ist eine Verbesserung der Ernährungssituation durch prothetische Rehabilitation mit Implantaten in Kombination mit einer Ernährungsberatung zu erreichen?

Es handelt sich um eine rein explorative Studie, deren Ergebnisse zur Planung einer folgenden konfirmatorischen Studie dienen sollen..

Biometric support care regarding methodology, Study-desin, randomization and evaluation is carried out by AG Medical Statistics of the Institute of Medical computer science at the University Hospital Giessen (Director : Dr. R.-H. Bödeker).

The primary target is related to the overall treatment (from the start of treatment t0 to t2 Recall last 12 months): Is an improvement of the nutritional situation through prosthetic rehabilitation to achieve with implants in combination with dietary advice ? The study is planned as a pilot study, whose results are used to plan a subsequent confirmatory study..
